# (4-Methoxy­benzoyl­meth­yl)triphenyl­phospho­nium trifluoro­methane­sulfonate

**DOI:** 10.1107/S1600536809000919

**Published:** 2009-01-14

**Authors:** Kazem Karami, Orhan Büyükgüngör

**Affiliations:** a Department of Chemistry, Isfahan University of Technology, Isfahan 84156/83111, Iran; b Department of Physics, Faculty of Arts and Sciences, Ondokuz Mayıs University, 55139 Samsun, Turkey

## Abstract

Colourless crystals of the title compound, C_27_H_24_O_2_P^+^·CF_3_SO_3_^−^, have been prepared by the addition of a solution of AgCF_3_SO_3_ in methanol to a solution of (4-methoxy­benzoyl­meth­yl)triphenyl­phospho­nium bromide in dry methanol. There are two crystallographically independent mol­ecules in the asymmetric unit. The crystal structure is stabilized by inter- and intra­molecular C—H⋯O hydrogen bonds and further stabilized by C—H⋯π inter­actions.

## Related literature

For background to phospho­rus ylides, see: Akkurt *et al.* (2008 ▶);; Kalyanasundari *et al.* (1995 ▶, 1999 ▶); Kolodiazhnyi (1996 ▶); Laavanya *et al.* (2001 ▶); Vicente *et al.* (1985 ▶). For the synthesis, see: Burmeister *et al.* (1973 ▶).
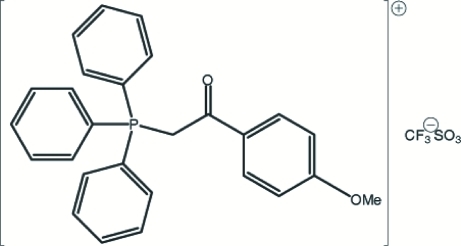


## Experimental

### 

#### Crystal data

C_27_H_24_O_2_P^+^·CF_3_O_3_S^−^
*M* *_r_* = 560.50
Monoclinic, 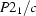

*a* = 10.6641 (5) Å
*b* = 20.2760 (12) Å
*c* = 25.0960 (11) Å
β = 96.539 (3)°
*V* = 5391.1 (5) Å^3^
*Z* = 8
Mo *K*α radiation
μ = 0.24 mm^−1^
*T* = 293 (2) K
0.67 × 0.33 × 0.14 mm

#### Data collection

Stoe IPDSII diffractometer
Absorption correction: integration (*X-RED32*: Stoe & Cie, 2002 ▶) *T*_min_ = 0.766, *T*_max_ = 0.902
75273 measured reflections
11185 independent reflections
6282 reflections with *I* > 2σ(*I*)
*R*_int_ = 0.082

#### Refinement

*R*[*F* ^2^ > 2σ(*F* ^2^)] = 0.079
*wR*(*F* ^2^) = 0.188
*S* = 1.07
11185 reflections
685 parameters
H-atom parameters constrained
Δρ_max_ = 0.46 e Å^−3^
Δρ_min_ = −0.29 e Å^−3^

### 

Data collection: *X-AREA* (Stoe & Cie, 2002 ▶); cell refinement: *X-AREA*; data reduction: *X-RED32* (Stoe & Cie, 2002 ▶); program(s) used to solve structure: *SIR97* (Altomare *et al.*, 1999 ▶); program(s) used to refine structure: *SHELXL97* (Sheldrick, 2008 ▶); molecular graphics: *ORTEP-3* (Farrugia, 1997 ▶); software used to prepare material for publication: *WinGX* (Farrugia, 1999 ▶).

## Supplementary Material

Crystal structure: contains datablocks I, global. DOI: 10.1107/S1600536809000919/at2703sup1.cif

Structure factors: contains datablocks I. DOI: 10.1107/S1600536809000919/at2703Isup2.hkl

Additional supplementary materials:  crystallographic information; 3D view; checkCIF report

## Figures and Tables

**Table 1:** Hydrogen-bond geometry (Å, °)

*D*—H⋯*A*	*D*—H	H⋯*A*	*D*⋯*A*	*D*—H⋯*A*
C19—H19*A*⋯O5	0.97	2.34	3.207 (6)	149
C19—H19*B*⋯O3^i^	0.97	2.54	3.487 (5)	166
C33—H33⋯O6^ii^	0.93	2.34	3.130 (6)	143
C45—H45⋯O3	0.93	2.52	3.071 (6)	118
C46—H46*A*⋯O1	0.97	2.48	3.417 (5)	162
C46—H46*B*⋯O10^iii^	0.97	2.19	3.137 (7)	165
C44—H44⋯*Cg*4^iv^	0.93	2.83	3.640 (6)	147

Symmetry codes: (i) 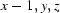; (ii) 
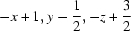
; (iii) 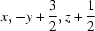; (iv) 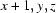. *Cg*4 is the centroid of the C21–C26 benzene ring.

**Fig. 1.**
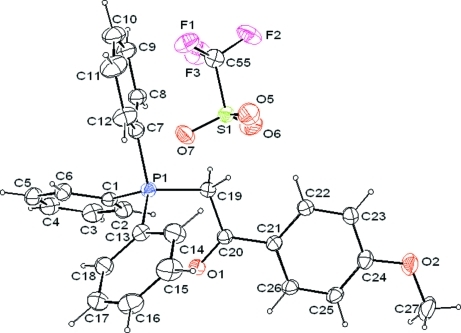
ORTEP plotting of one molecule in the asymmetric unit of the title compound (I) with the atom numbering scheme. Displacement ellipsoids for non-H atoms are drawn at the 30% probability level.

**Fig. 2.**
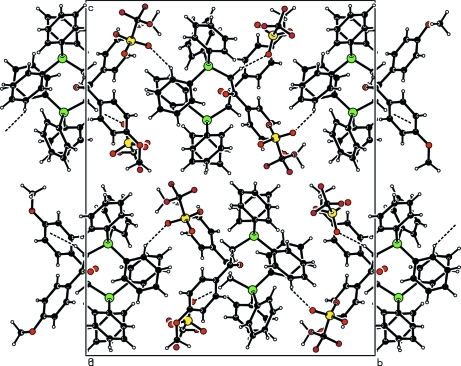
The packing and hydrogen bonding interactions of (I), viewed down a axis.

**Fig. 3.**
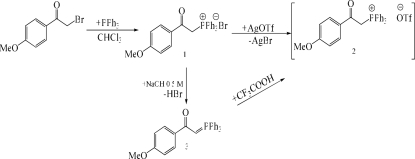
The formation of the title compound.

## supplementary crystallographic information

### Comment

Phosphorus ylide have been increasingly studied over the past decade because of their interesting structure. the utilies of metalated phosphorus ylides in synthetic chemistry have been well documented (Kolodiazhnyi *et al.*, 1996). The α-keto-stabilized phosphorus ylides R_3_P═CHCOR show interesting properties such as high stability and ambidentate character as ligands (C-versus O-coordination) (Vicente *et al.*, 1985; Kalyanasundari *et al.*, 1995; Laavanya *et al.*, 2001). Phosphorus ylides are known to demonstrate rich coordination chemistry. One of the significance aspects of our work is taking knowledge about preferred coordination modes of FBPPY, CBPPY, BrBPPY, and MOBPPY to the Hg and Pd metals (Akkurt *et al.*, 2008). The phosphonium salt (I) (Fig. 1) was synthesized according to sequence mentioned in Scheme 2 (Burmeister *et al.*, 1973).

In this communication we have reported the preparations and structures of new tri-folourosulfonate phosphonium ylides.

In the asymmetric unit of (I), there are two crystallographically independent molecules (Fig. 1). A comparison of the bond lengths and bond angles in (I) shows that the phosphonium cation as a ligand is electrostatically under the influence of an anionic part of OTf in the unit cells. X-ray structural analysis established that Fig. 1 contains of discrete [CH_3_OC_6_H_4_COCH_2_PPh_3_]^+^ cations and OTf^-^ anions in a 1:1 ratio. An ORTEP plot (Fig. 1) and crystal packing view (Fig. 2) show that the double tetrahedral OTf unit is formed by sharing one tetrahedral edge, and possesses approximate C_3_V symmetry. These units are held together by electrostatic forces. In the fact, the crystal of the phosphonium (I) shows a close association between the OTf^-^ and the inner sphere, with O···H distances of 3.07 (16) and 3.487 (5) Å (see Fig. 2, Table 1).

Comparision of the bond lengths and bond angles within the above crystal show that the phosphonium as a ligand is electrostatically under the influence of an anionic part of trifloro solfonate in the unit cells (for instance, the bond lengths C21—C20, O1—C20, C20—C19, C19—P1 and P1—C1 and bond angles C19—P1—C1, O1—C20—C21 are 1.471 (5), 1.226 (5), 1.517 (6), 1.798 (4) and 1.797 (4) Å and 113.17 (19) and 120.4 (4)° for the title compound and 1.493 (9), 1.212 (9), 1.491 (10), 1787 (6) and 1.806 (8) Å and 122.2 (4)° for the phosphorane molecule (Laavanya *et al.*, 2001).

The P—C bond length [1.727 (2) Å] is shorter than the other P—C bonds and longer than the equivalent bond lengths of 1.66 Å, reported for methylenetriphenylphosphorane, which shows partial double-bond character for these two bonds.

The crystal structure is stabilized by C—H···O hydrogen bonds and further by C—H···π interactions.

### Experimental

The title compound was obtained from reaction between (I) and AgOTf in dry methanol in a 1:1 molar ratio and under stirring for 12 h. The product was washed sveral times with dry diethyl ether and dried in a vacuum. Colourless plate crystals of C_27_H_24_F_3_O_5_P appeared by addition of dry diethyl ether to a chlroform solution. The crystal structure consists of discrete [CH_3_OC_6_H_4_COCH_2_PPh_3_]+, cations and OTf^-^ anions in a 1:1 ratio. The obtained crystals of (I) are highly air stable and resistant against moisture.

### Refinement

All H atoms were positioned geometrically and were treated as riding, with C—H distances in the range 0.93-0.97 Å, and with *U*_iso_(H) = 1.2*U*_eq_(C) for aromatic and methylene H atoms and 1.5*U*_eq_(C) for methyl H atoms.

### Crystal data

**Table:**

C_27_H_24_O_2_P^+^·CF_3_O_3_S^−^	*F*(000) = 2320
*M**_r_* = 560.50	*D*_x_ = 1.381 Mg m^−^^3^*D*_m_ = 1.381 Mg m^−^^3^*D*_m_ measured by not measured
Monoclinic, *P*2_1_/*c*	Melting point: 296 K
Hall symbol: -P 2ybc	Mo *K*α radiation, λ = 0.71073 Å
*a* = 10.6641 (5) Å	Cell parameters from 685 reflections
*b* = 20.2760 (12) Å	θ = 1–26.5°
*c* = 25.0960 (11) Å	µ = 0.24 mm^−^^1^
β = 96.539 (3)°	*T* = 293 K
*V* = 5391.1 (5) Å^3^	Prism, yellow
*Z* = 8	0.67 × 0.33 × 0.14 mm

### Data collection

**Table:**

Stoe IPDSII diffractometer	11185 independent reflections
Radiation source: fine-focfine-focus us sealed tube	6282 reflections with *I* > 2σ(*I*)
graphite	*R*_int_ = 0.082
Detector resolution: 6.67 pixels mm^-1^	θ_max_ = 26.5°, θ_min_ = 1.3°
rotation scans	*h* = −13→13
Absorption correction: integration (*X-RED32*: Stoe & Cie, 2002)	*k* = −25→25
*T*_min_ = 0.766, *T*_max_ = 0.902	*l* = −31→31
75273 measured reflections	

### Refinement

**Table:**

Refinement on *F*^2^	Primary atom site location: structure-invariant direct methods
Least-squares matrix: full	Secondary atom site location: difference Fourier map
*R*[*F*^2^ > 2σ(*F*^2^)] = 0.079	Hydrogen site location: inferred from neighbouring sites
*wR*(*F*^2^) = 0.188	H-atom parameters constrained
*S* = 1.07	*w* = 1/[σ^2^(*F*_o_^2^) + (0.0814*P*)^2^] where *P* = (*F*_o_^2^ + 2*F*_c_^2^)/3
11185 reflections	(Δ/σ)_max_ < 0.001
685 parameters	Δρ_max_ = 0.46 e Å^−^^3^
0 restraints	Δρ_min_ = −0.29 e Å^−^^3^
0 constraints	

### Special details

**Table:**

Geometry. All e.s.d.'s (except the e.s.d. in the dihedral angle between two l.s. planes) are estimated using the full covariance matrix. The cell e.s.d.'s are taken into account individually in the estimation of e.s.d.'s in distances, angles and torsion angles; correlations between e.s.d.'s in cell parameters are only used when they are defined by crystal symmetry. An approximate (isotropic) treatment of cell e.s.d.'s is used for estimating e.s.d.'s involving l.s. planes.
Refinement. Refinement of *F*^2^ against ALL reflections. The weighted *R*-factor *wR* and goodness of fit *S* are based on *F*^2^, conventional *R*-factors *R* are based on *F*, with *F* set to zero for negative *F*^2^. The threshold expression of *F*^2^ > σ(*F*^2^) is used only for calculating *R*-factors(gt) *etc*. and is not relevant to the choice of reflections for refinement. *R*-factors based on *F*^2^ are statistically about twice as large as those based on *F*, and *R*- factors based on ALL data will be even larger.

### Fractional atomic coordinates and isotropic or equivalent isotropic displacement parameters (Å^2^)

**Table:**

	*x*	*y*	*z*	*U*_iso_*/*U*_eq_	
C1	0.2371 (3)	0.4197 (2)	0.62037 (15)	0.0498 (9)	
C2	0.3253 (4)	0.4694 (2)	0.62123 (18)	0.0650 (11)	
H2	0.3261	0.5035	0.6461	0.078*	
C3	0.4130 (4)	0.4678 (3)	0.5844 (2)	0.0803 (14)	
H3	0.4732	0.5010	0.5845	0.096*	
C4	0.4111 (4)	0.4177 (3)	0.54798 (19)	0.0798 (15)	
H4	0.4699	0.4175	0.5233	0.096*	
C5	0.3252 (4)	0.3682 (3)	0.54703 (17)	0.0723 (13)	
H5	0.3262	0.3339	0.5225	0.087*	
C6	0.2369 (4)	0.3695 (2)	0.58279 (16)	0.0602 (11)	
H6	0.1763	0.3364	0.5818	0.072*	
C7	−0.0308 (4)	0.4156 (2)	0.61744 (15)	0.0528 (9)	
C8	−0.0458 (4)	0.4641 (2)	0.57783 (16)	0.0622 (11)	
H8	0.0151	0.4968	0.5769	0.075*	
C9	−0.1508 (4)	0.4638 (3)	0.53982 (18)	0.0742 (13)	
H9	−0.1609	0.4963	0.5135	0.089*	
C10	−0.2396 (5)	0.4153 (3)	0.5414 (2)	0.0873 (16)	
H10	−0.3085	0.4140	0.5151	0.105*	
C11	−0.2280 (5)	0.3693 (3)	0.5808 (2)	0.1002 (19)	
H11	−0.2912	0.3380	0.5823	0.120*	
C12	−0.1216 (4)	0.3684 (3)	0.6196 (2)	0.0782 (14)	
H12	−0.1133	0.3362	0.6462	0.094*	
C13	0.1237 (4)	0.3494 (2)	0.70769 (15)	0.0543 (10)	
C14	0.0411 (5)	0.3450 (3)	0.74678 (19)	0.0782 (14)	
H14	−0.0195	0.3774	0.7495	0.094*	
C15	0.0505 (5)	0.2920 (3)	0.7812 (2)	0.0906 (16)	
H15	−0.0046	0.2887	0.8073	0.109*	
C16	0.1381 (5)	0.2446 (3)	0.7779 (2)	0.0836 (15)	
H16	0.1435	0.2094	0.8018	0.100*	
C17	0.2186 (5)	0.2481 (2)	0.7395 (2)	0.0775 (13)	
H17	0.2774	0.2147	0.7368	0.093*	
C18	0.2133 (4)	0.3009 (2)	0.70465 (17)	0.0642 (11)	
H18	0.2699	0.3038	0.6792	0.077*	
C19	0.1036 (4)	0.4932 (2)	0.70104 (15)	0.0539 (10)	
H19A	0.1028	0.5307	0.6770	0.065*	
H19B	0.0247	0.4935	0.7169	0.065*	
C20	0.2119 (4)	0.5012 (2)	0.74527 (15)	0.0503 (9)	
C21	0.1997 (3)	0.55009 (19)	0.78769 (15)	0.0495 (9)	
C22	0.1026 (4)	0.5967 (2)	0.78495 (17)	0.0592 (10)	
H22	0.0417	0.5973	0.7553	0.071*	
C23	0.0962 (4)	0.6414 (2)	0.82528 (17)	0.0652 (11)	
H23	0.0316	0.6725	0.8228	0.078*	
C24	0.1854 (4)	0.6404 (2)	0.86965 (17)	0.0637 (11)	
C25	0.2826 (4)	0.5952 (2)	0.87317 (16)	0.0637 (12)	
H25	0.3433	0.5948	0.9029	0.076*	
C26	0.2887 (4)	0.5508 (2)	0.83254 (16)	0.0574 (10)	
H26	0.3542	0.5202	0.8350	0.069*	
C27	0.2565 (6)	0.6850 (4)	0.9558 (2)	0.119 (2)	
H27A	0.2362	0.7201	0.9790	0.179*	
H27B	0.3415	0.6903	0.9473	0.179*	
H27C	0.2489	0.6434	0.9735	0.179*	
C28	0.6457 (3)	0.3595 (2)	0.76582 (15)	0.0513 (9)	
C29	0.5704 (4)	0.3669 (2)	0.71752 (17)	0.0659 (12)	
H29	0.5308	0.4070	0.7090	0.079*	
C30	0.5537 (5)	0.3152 (3)	0.68215 (19)	0.0781 (14)	
H30	0.5015	0.3201	0.6501	0.094*	
C31	0.6134 (5)	0.2572 (3)	0.6939 (2)	0.0835 (15)	
H31	0.6030	0.2224	0.6696	0.100*	
C32	0.6883 (5)	0.2494 (3)	0.7409 (2)	0.0913 (16)	
H32	0.7285	0.2092	0.7486	0.110*	
C33	0.7055 (4)	0.3002 (2)	0.77731 (18)	0.0702 (12)	
H33	0.7571	0.2946	0.8094	0.084*	
C34	0.5360 (4)	0.40709 (19)	0.85901 (15)	0.0517 (9)	
C35	0.4350 (4)	0.3661 (2)	0.84246 (18)	0.0716 (13)	
H35	0.4278	0.3472	0.8085	0.086*	
C36	0.3453 (5)	0.3534 (3)	0.8767 (2)	0.1001 (19)	
H36	0.2769	0.3262	0.8658	0.120*	
C37	0.3577 (6)	0.3812 (4)	0.9268 (3)	0.104 (2)	
H37	0.2983	0.3717	0.9501	0.125*	
C38	0.4554 (6)	0.4223 (3)	0.9431 (2)	0.0998 (18)	
H38	0.4620	0.4412	0.9770	0.120*	
C39	0.5447 (5)	0.4357 (2)	0.90883 (18)	0.0732 (13)	
H39	0.6110	0.4643	0.9196	0.088*	
C40	0.8025 (4)	0.4269 (2)	0.85787 (16)	0.0548 (10)	
C41	0.8310 (4)	0.3769 (2)	0.89511 (18)	0.0698 (12)	
H41	0.7763	0.3413	0.8965	0.084*	
C42	0.9406 (5)	0.3798 (3)	0.9301 (2)	0.0839 (15)	
H42	0.9601	0.3459	0.9546	0.101*	
C43	1.0204 (5)	0.4323 (3)	0.9288 (2)	0.0912 (17)	
H43	1.0938	0.4343	0.9526	0.109*	
C44	0.9928 (5)	0.4816 (3)	0.8928 (2)	0.0897 (16)	
H44	1.0476	0.5172	0.8920	0.108*	
C45	0.8839 (4)	0.4793 (2)	0.8573 (2)	0.0727 (13)	
H45	0.8656	0.5134	0.8329	0.087*	
C46	0.6179 (4)	0.50060 (19)	0.78234 (16)	0.0514 (9)	
H46A	0.5295	0.5006	0.7679	0.062*	
H46B	0.6304	0.5360	0.8084	0.062*	
C47	0.6978 (4)	0.5137 (2)	0.73732 (16)	0.0534 (10)	
C48	0.6536 (3)	0.56138 (19)	0.69497 (15)	0.0495 (9)	
C49	0.5466 (4)	0.6000 (2)	0.69589 (17)	0.0607 (11)	
H49	0.5008	0.5974	0.7252	0.073*	
C50	0.5068 (4)	0.6422 (2)	0.65444 (19)	0.0709 (12)	
H50	0.4345	0.6676	0.6556	0.085*	
C51	0.5753 (4)	0.6464 (2)	0.61101 (17)	0.0660 (12)	
C52	0.6829 (5)	0.6085 (3)	0.6100 (2)	0.0819 (15)	
H52	0.7296	0.6115	0.5810	0.098*	
C53	0.7206 (4)	0.5672 (2)	0.65113 (18)	0.0676 (12)	
H53	0.7932	0.5421	0.6499	0.081*	
C54	0.4351 (6)	0.7247 (3)	0.5649 (2)	0.110 (2)	
H54A	0.4263	0.7492	0.5320	0.165*	
H54B	0.4419	0.7546	0.5947	0.165*	
H54C	0.3625	0.6970	0.5663	0.165*	
C55	−0.0022 (5)	0.6781 (3)	0.5545 (2)	0.0789 (14)	
C56	0.8050 (6)	0.8346 (3)	0.4279 (3)	0.0893 (16)	
F1	−0.0619 (3)	0.6251 (2)	0.53358 (14)	0.1184 (12)	
F2	−0.0878 (3)	0.72038 (18)	0.56646 (15)	0.1195 (12)	
F3	0.0579 (4)	0.7046 (2)	0.51722 (14)	0.1332 (14)	
F4	0.8159 (4)	0.8128 (3)	0.47665 (15)	0.1514 (17)	
F5	0.8797 (5)	0.8827 (3)	0.4279 (3)	0.211 (3)	
F6	0.8564 (4)	0.7876 (2)	0.40148 (19)	0.1530 (16)	
O1	0.3065 (3)	0.46701 (15)	0.74453 (11)	0.0653 (8)	
O2	0.1719 (3)	0.68647 (18)	0.90766 (14)	0.0893 (11)	
O3	0.7972 (3)	0.48392 (16)	0.73638 (12)	0.0718 (9)	
O4	0.5451 (4)	0.68504 (19)	0.56762 (13)	0.0946 (11)	
O5	0.0177 (4)	0.6331 (2)	0.64958 (14)	0.1079 (13)	
O6	0.1662 (4)	0.71576 (19)	0.6272 (2)	0.1266 (16)	
O7	0.1802 (3)	0.60495 (18)	0.59502 (17)	0.0979 (12)	
O8	0.5820 (5)	0.7954 (3)	0.4125 (3)	0.183 (3)	
O9	0.6181 (6)	0.9064 (3)	0.4313 (2)	0.157 (2)	
O10	0.6530 (6)	0.8681 (3)	0.34844 (17)	0.154 (2)	
P1	0.11235 (9)	0.41867 (5)	0.66285 (4)	0.0478 (3)	
P2	0.65496 (9)	0.42347 (5)	0.81549 (4)	0.0482 (3)	
S1	0.10381 (12)	0.65597 (6)	0.61330 (5)	0.0681 (3)	
S2	0.64543 (14)	0.85241 (7)	0.40166 (5)	0.0844 (4)	

### Atomic displacement parameters (Å^2^)

**Table:**

	*U*^11^	*U*^22^	*U*^33^	*U*^12^	*U*^13^	*U*^23^
C1	0.043 (2)	0.057 (2)	0.048 (2)	0.0017 (19)	0.0011 (16)	0.0038 (19)
C2	0.057 (3)	0.072 (3)	0.066 (3)	−0.004 (2)	0.007 (2)	0.002 (2)
C3	0.056 (3)	0.102 (4)	0.084 (3)	−0.018 (3)	0.015 (2)	0.012 (3)
C4	0.056 (3)	0.127 (5)	0.059 (3)	0.010 (3)	0.017 (2)	0.010 (3)
C5	0.066 (3)	0.100 (4)	0.052 (2)	0.010 (3)	0.013 (2)	−0.003 (2)
C6	0.064 (3)	0.066 (3)	0.052 (2)	−0.002 (2)	0.011 (2)	−0.002 (2)
C7	0.049 (2)	0.059 (3)	0.051 (2)	−0.002 (2)	0.0066 (17)	−0.0054 (19)
C8	0.053 (2)	0.071 (3)	0.061 (3)	0.001 (2)	−0.0010 (19)	0.001 (2)
C9	0.063 (3)	0.099 (4)	0.057 (3)	0.009 (3)	−0.006 (2)	−0.002 (2)
C10	0.052 (3)	0.137 (5)	0.071 (3)	−0.001 (3)	−0.005 (2)	−0.005 (3)
C11	0.062 (3)	0.137 (5)	0.099 (4)	−0.040 (3)	−0.004 (3)	−0.006 (4)
C12	0.068 (3)	0.090 (4)	0.074 (3)	−0.022 (3)	−0.001 (2)	0.013 (3)
C13	0.053 (2)	0.060 (3)	0.050 (2)	−0.004 (2)	0.0040 (18)	0.0005 (19)
C14	0.071 (3)	0.096 (4)	0.071 (3)	0.013 (3)	0.021 (2)	0.020 (3)
C15	0.083 (4)	0.112 (5)	0.081 (3)	−0.005 (3)	0.023 (3)	0.031 (3)
C16	0.096 (4)	0.077 (4)	0.076 (3)	−0.016 (3)	−0.001 (3)	0.027 (3)
C17	0.088 (3)	0.068 (3)	0.075 (3)	0.013 (3)	0.001 (3)	0.011 (3)
C18	0.066 (3)	0.067 (3)	0.061 (3)	0.005 (2)	0.011 (2)	0.004 (2)
C19	0.048 (2)	0.059 (2)	0.053 (2)	0.0056 (19)	0.0005 (18)	−0.0051 (19)
C20	0.046 (2)	0.055 (2)	0.049 (2)	−0.0009 (19)	0.0031 (17)	0.0014 (18)
C21	0.047 (2)	0.053 (2)	0.048 (2)	−0.0055 (18)	0.0044 (16)	0.0001 (18)
C22	0.059 (2)	0.059 (3)	0.058 (2)	0.004 (2)	−0.0016 (19)	−0.004 (2)
C23	0.065 (3)	0.061 (3)	0.069 (3)	0.007 (2)	0.004 (2)	−0.012 (2)
C24	0.065 (3)	0.065 (3)	0.063 (3)	−0.010 (2)	0.013 (2)	−0.012 (2)
C25	0.050 (2)	0.087 (3)	0.052 (2)	−0.006 (2)	−0.0022 (18)	−0.010 (2)
C26	0.046 (2)	0.071 (3)	0.054 (2)	0.001 (2)	−0.0003 (18)	−0.001 (2)
C27	0.111 (5)	0.167 (7)	0.077 (4)	0.003 (4)	−0.001 (3)	−0.065 (4)
C28	0.041 (2)	0.057 (2)	0.056 (2)	0.0049 (18)	0.0039 (17)	0.0036 (19)
C29	0.064 (3)	0.071 (3)	0.059 (3)	0.015 (2)	−0.006 (2)	−0.003 (2)
C30	0.071 (3)	0.098 (4)	0.062 (3)	0.011 (3)	−0.003 (2)	−0.013 (3)
C31	0.082 (3)	0.084 (4)	0.085 (4)	0.005 (3)	0.007 (3)	−0.031 (3)
C32	0.104 (4)	0.074 (3)	0.093 (4)	0.032 (3)	−0.003 (3)	−0.013 (3)
C33	0.079 (3)	0.069 (3)	0.059 (3)	0.021 (3)	−0.005 (2)	−0.006 (2)
C34	0.051 (2)	0.051 (2)	0.054 (2)	0.0043 (18)	0.0060 (17)	0.0061 (18)
C35	0.060 (3)	0.094 (4)	0.060 (3)	−0.013 (3)	0.006 (2)	0.006 (2)
C36	0.067 (3)	0.139 (5)	0.098 (4)	−0.030 (3)	0.024 (3)	0.001 (4)
C37	0.082 (4)	0.144 (6)	0.095 (4)	0.003 (4)	0.043 (3)	0.019 (4)
C38	0.109 (5)	0.128 (5)	0.068 (3)	0.003 (4)	0.037 (3)	−0.003 (3)
C39	0.077 (3)	0.080 (3)	0.065 (3)	−0.008 (3)	0.020 (2)	−0.004 (2)
C40	0.050 (2)	0.054 (2)	0.059 (2)	0.002 (2)	0.0004 (18)	0.0033 (19)
C41	0.067 (3)	0.058 (3)	0.080 (3)	−0.003 (2)	−0.014 (2)	0.010 (2)
C42	0.081 (3)	0.076 (3)	0.088 (3)	0.013 (3)	−0.024 (3)	0.018 (3)
C43	0.056 (3)	0.101 (4)	0.109 (4)	−0.008 (3)	−0.025 (3)	0.012 (3)
C44	0.064 (3)	0.094 (4)	0.105 (4)	−0.025 (3)	−0.015 (3)	0.017 (3)
C45	0.063 (3)	0.069 (3)	0.083 (3)	−0.012 (2)	−0.005 (2)	0.012 (2)
C46	0.047 (2)	0.052 (2)	0.055 (2)	0.0065 (18)	0.0074 (17)	0.0061 (18)
C47	0.048 (2)	0.052 (2)	0.061 (2)	0.0030 (19)	0.0104 (18)	0.0085 (19)
C48	0.045 (2)	0.049 (2)	0.055 (2)	0.0013 (17)	0.0077 (17)	0.0053 (17)
C49	0.062 (3)	0.063 (3)	0.059 (2)	0.009 (2)	0.016 (2)	0.013 (2)
C50	0.068 (3)	0.069 (3)	0.076 (3)	0.017 (2)	0.010 (2)	0.014 (2)
C51	0.077 (3)	0.063 (3)	0.057 (3)	0.001 (2)	0.002 (2)	0.018 (2)
C52	0.072 (3)	0.106 (4)	0.071 (3)	0.005 (3)	0.021 (2)	0.026 (3)
C53	0.051 (2)	0.086 (3)	0.067 (3)	0.010 (2)	0.011 (2)	0.020 (2)
C54	0.113 (5)	0.102 (5)	0.107 (4)	0.030 (4)	−0.019 (4)	0.036 (4)
C55	0.080 (3)	0.076 (3)	0.082 (3)	−0.003 (3)	0.013 (3)	0.012 (3)
C56	0.105 (4)	0.071 (4)	0.093 (4)	−0.008 (3)	0.013 (3)	−0.011 (3)
F1	0.107 (2)	0.135 (3)	0.104 (2)	−0.025 (2)	−0.0280 (19)	−0.013 (2)
F2	0.102 (2)	0.116 (3)	0.140 (3)	0.044 (2)	0.009 (2)	0.038 (2)
F3	0.146 (3)	0.164 (4)	0.095 (2)	0.002 (3)	0.037 (2)	0.054 (2)
F4	0.140 (3)	0.216 (5)	0.090 (3)	0.046 (3)	−0.023 (2)	0.014 (3)
F5	0.134 (4)	0.124 (4)	0.361 (9)	−0.039 (3)	−0.029 (5)	0.026 (5)
F6	0.148 (4)	0.148 (4)	0.164 (4)	0.042 (3)	0.024 (3)	−0.029 (3)
O1	0.0527 (16)	0.079 (2)	0.0620 (17)	0.0156 (15)	−0.0035 (13)	−0.0125 (15)
O2	0.084 (2)	0.097 (3)	0.086 (2)	−0.001 (2)	0.0076 (19)	−0.042 (2)
O3	0.0541 (17)	0.087 (2)	0.077 (2)	0.0193 (16)	0.0190 (15)	0.0247 (17)
O4	0.106 (3)	0.105 (3)	0.072 (2)	0.026 (2)	0.0070 (19)	0.039 (2)
O5	0.134 (3)	0.120 (3)	0.074 (2)	0.020 (3)	0.028 (2)	0.029 (2)
O6	0.131 (3)	0.079 (3)	0.157 (4)	−0.016 (2)	−0.040 (3)	−0.034 (3)
O7	0.082 (2)	0.078 (2)	0.129 (3)	0.023 (2)	−0.005 (2)	−0.019 (2)
O8	0.132 (4)	0.163 (5)	0.236 (6)	−0.068 (4)	−0.057 (4)	0.103 (5)
O9	0.175 (5)	0.148 (4)	0.147 (4)	0.073 (4)	0.009 (4)	−0.039 (4)
O10	0.218 (6)	0.161 (5)	0.079 (3)	0.029 (4)	−0.004 (3)	0.048 (3)
P1	0.0438 (5)	0.0532 (6)	0.0460 (5)	0.0001 (5)	0.0034 (4)	−0.0011 (5)
P2	0.0453 (5)	0.0489 (6)	0.0502 (5)	0.0029 (5)	0.0047 (4)	0.0062 (5)
S1	0.0765 (8)	0.0559 (7)	0.0675 (7)	0.0042 (6)	−0.0102 (6)	−0.0071 (6)
S2	0.0943 (10)	0.0832 (10)	0.0727 (8)	0.0104 (8)	−0.0036 (7)	0.0098 (7)

### Geometric parameters (Å, °)

**Table:**

C1—C2	1.378 (6)	C31—C32	1.357 (7)
C1—C6	1.387 (6)	C31—H31	0.9300
C1—P1	1.797 (4)	C32—C33	1.376 (7)
C2—C3	1.389 (6)	C32—H32	0.9300
C2—H2	0.9300	C33—H33	0.9300
C3—C4	1.365 (7)	C34—C39	1.372 (6)
C3—H3	0.9300	C34—C35	1.387 (6)
C4—C5	1.357 (7)	C34—P2	1.796 (4)
C4—H4	0.9300	C35—C36	1.381 (7)
C5—C6	1.373 (6)	C35—H35	0.9300
C5—H5	0.9300	C36—C37	1.370 (8)
C6—H6	0.9300	C36—H36	0.9300
C7—C12	1.368 (6)	C37—C38	1.360 (9)
C7—C8	1.394 (6)	C37—H37	0.9300
C7—P1	1.799 (4)	C38—C39	1.380 (7)
C8—C9	1.386 (6)	C38—H38	0.9300
C8—H8	0.9300	C39—H39	0.9300
C9—C10	1.369 (7)	C40—C45	1.373 (6)
C9—H9	0.9300	C40—C41	1.390 (6)
C10—C11	1.355 (8)	C40—P2	1.797 (4)
C10—H10	0.9300	C41—C42	1.380 (6)
C11—C12	1.409 (7)	C41—H41	0.9300
C11—H11	0.9300	C42—C43	1.366 (7)
C12—H12	0.9300	C42—H42	0.9300
C13—C18	1.379 (6)	C43—C44	1.356 (7)
C13—C14	1.394 (6)	C43—H43	0.9300
C13—P1	1.795 (4)	C44—C45	1.382 (6)
C14—C15	1.376 (7)	C44—H44	0.9300
C14—H14	0.9300	C45—H45	0.9300
C15—C16	1.349 (7)	C46—C47	1.514 (5)
C15—H15	0.9300	C46—P2	1.794 (4)
C16—C17	1.364 (7)	C46—H46A	0.9700
C16—H16	0.9300	C46—H46B	0.9700
C17—C18	1.379 (6)	C47—O3	1.223 (5)
C17—H17	0.9300	C47—C48	1.473 (5)
C18—H18	0.9300	C48—C53	1.384 (6)
C19—C20	1.517 (5)	C48—C49	1.386 (5)
C19—P1	1.798 (4)	C49—C50	1.376 (6)
C19—H19A	0.9700	C49—H49	0.9300
C19—H19B	0.9700	C50—C51	1.382 (6)
C20—O1	1.226 (4)	C50—H50	0.9300
C20—C21	1.471 (5)	C51—O4	1.350 (5)
C21—C26	1.387 (5)	C51—C52	1.384 (7)
C21—C22	1.398 (6)	C52—C53	1.354 (6)
C22—C23	1.366 (6)	C52—H52	0.9300
C22—H22	0.9300	C53—H53	0.9300
C23—C24	1.380 (6)	C54—O4	1.417 (6)
C23—H23	0.9300	C54—H54A	0.9600
C24—O2	1.355 (5)	C54—H54B	0.9600
C24—C25	1.379 (6)	C54—H54C	0.9600
C25—C26	1.367 (6)	C55—F3	1.308 (6)
C25—H25	0.9300	C55—F2	1.312 (6)
C26—H26	0.9300	C55—F1	1.326 (6)
C27—O2	1.423 (6)	C55—S1	1.811 (5)
C27—H27A	0.9600	C56—F5	1.260 (7)
C27—H27B	0.9600	C56—F4	1.293 (7)
C27—H27C	0.9600	C56—F6	1.317 (6)
C28—C33	1.376 (6)	C56—S2	1.791 (7)
C28—C29	1.384 (5)	O5—S1	1.441 (4)
C28—P2	1.794 (4)	O6—S1	1.408 (4)
C29—C30	1.371 (6)	O7—S1	1.424 (4)
C29—H29	0.9300	O8—S2	1.381 (5)
C30—C31	1.355 (7)	O9—S2	1.374 (5)
C30—H30	0.9300	O10—S2	1.384 (4)
			
C2—C1—C6	119.7 (4)	C39—C34—P2	119.8 (3)
C2—C1—P1	123.3 (3)	C35—C34—P2	120.5 (3)
C6—C1—P1	116.8 (3)	C36—C35—C34	119.7 (5)
C1—C2—C3	118.9 (5)	C36—C35—H35	120.2
C1—C2—H2	120.6	C34—C35—H35	120.2
C3—C2—H2	120.6	C37—C36—C35	119.5 (5)
C4—C3—C2	120.2 (5)	C37—C36—H36	120.2
C4—C3—H3	119.9	C35—C36—H36	120.2
C2—C3—H3	119.9	C38—C37—C36	121.2 (5)
C5—C4—C3	121.4 (4)	C38—C37—H37	119.4
C5—C4—H4	119.3	C36—C37—H37	119.4
C3—C4—H4	119.3	C37—C38—C39	119.5 (5)
C4—C5—C6	119.1 (5)	C37—C38—H38	120.2
C4—C5—H5	120.5	C39—C38—H38	120.2
C6—C5—H5	120.5	C34—C39—C38	120.3 (5)
C5—C6—C1	120.7 (4)	C34—C39—H39	119.9
C5—C6—H6	119.6	C38—C39—H39	119.9
C1—C6—H6	119.6	C45—C40—C41	118.8 (4)
C12—C7—C8	119.9 (4)	C45—C40—P2	122.4 (3)
C12—C7—P1	123.5 (3)	C41—C40—P2	118.7 (3)
C8—C7—P1	116.6 (3)	C42—C41—C40	120.1 (4)
C9—C8—C7	120.4 (4)	C42—C41—H41	120.0
C9—C8—H8	119.8	C40—C41—H41	120.0
C7—C8—H8	119.8	C43—C42—C41	120.2 (5)
C10—C9—C8	119.4 (5)	C43—C42—H42	119.9
C10—C9—H9	120.3	C41—C42—H42	119.9
C8—C9—H9	120.3	C44—C43—C42	120.1 (4)
C11—C10—C9	120.6 (5)	C44—C43—H43	119.9
C11—C10—H10	119.7	C42—C43—H43	119.9
C9—C10—H10	119.7	C43—C44—C45	120.5 (5)
C10—C11—C12	120.9 (5)	C43—C44—H44	119.7
C10—C11—H11	119.5	C45—C44—H44	119.7
C12—C11—H11	119.5	C40—C45—C44	120.3 (4)
C7—C12—C11	118.7 (5)	C40—C45—H45	119.8
C7—C12—H12	120.6	C44—C45—H45	119.8
C11—C12—H12	120.6	C47—C46—P2	112.7 (3)
C18—C13—C14	119.4 (4)	C47—C46—H46A	109.0
C18—C13—P1	121.4 (3)	P2—C46—H46A	109.0
C14—C13—P1	119.2 (3)	C47—C46—H46B	109.0
C15—C14—C13	119.0 (5)	P2—C46—H46B	109.0
C15—C14—H14	120.5	H46A—C46—H46B	107.8
C13—C14—H14	120.5	O3—C47—C48	121.1 (4)
C16—C15—C14	121.2 (5)	O3—C47—C46	119.3 (3)
C16—C15—H15	119.4	C48—C47—C46	119.5 (3)
C14—C15—H15	119.4	C53—C48—C49	117.8 (4)
C15—C16—C17	120.2 (5)	C53—C48—C47	118.5 (4)
C15—C16—H16	119.9	C49—C48—C47	123.7 (4)
C17—C16—H16	119.9	C50—C49—C48	121.5 (4)
C16—C17—C18	120.3 (5)	C50—C49—H49	119.2
C16—C17—H17	119.9	C48—C49—H49	119.2
C18—C17—H17	119.9	C49—C50—C51	119.3 (4)
C17—C18—C13	119.9 (4)	C49—C50—H50	120.4
C17—C18—H18	120.1	C51—C50—H50	120.4
C13—C18—H18	120.1	O4—C51—C50	124.9 (4)
C20—C19—P1	113.4 (3)	O4—C51—C52	115.5 (4)
C20—C19—H19A	108.9	C50—C51—C52	119.6 (4)
P1—C19—H19A	108.9	C53—C52—C51	120.3 (4)
C20—C19—H19B	108.9	C53—C52—H52	119.8
P1—C19—H19B	108.9	C51—C52—H52	119.8
H19A—C19—H19B	107.7	C52—C53—C48	121.5 (4)
O1—C20—C21	122.2 (3)	C52—C53—H53	119.2
O1—C20—C19	119.2 (3)	C48—C53—H53	119.2
C21—C20—C19	118.6 (3)	O4—C54—H54A	109.5
C26—C21—C22	117.9 (4)	O4—C54—H54B	109.5
C26—C21—C20	119.0 (4)	H54A—C54—H54B	109.5
C22—C21—C20	123.1 (3)	O4—C54—H54C	109.5
C23—C22—C21	120.8 (4)	H54A—C54—H54C	109.5
C23—C22—H22	119.6	H54B—C54—H54C	109.5
C21—C22—H22	119.6	F3—C55—F2	107.8 (5)
C22—C23—C24	120.0 (4)	F3—C55—F1	107.7 (5)
C22—C23—H23	120.0	F2—C55—F1	107.8 (5)
C24—C23—H23	120.0	F3—C55—S1	111.9 (4)
O2—C24—C25	123.6 (4)	F2—C55—S1	111.1 (4)
O2—C24—C23	116.0 (4)	F1—C55—S1	110.4 (4)
C25—C24—C23	120.4 (4)	F5—C56—F4	106.1 (6)
C26—C25—C24	119.3 (4)	F5—C56—F6	105.2 (6)
C26—C25—H25	120.3	F4—C56—F6	103.4 (5)
C24—C25—H25	120.3	F5—C56—S2	114.7 (5)
C25—C26—C21	121.7 (4)	F4—C56—S2	113.3 (5)
C25—C26—H26	119.2	F6—C56—S2	113.2 (4)
C21—C26—H26	119.2	C24—O2—C27	118.3 (4)
O2—C27—H27A	109.5	C51—O4—C54	119.2 (4)
O2—C27—H27B	109.5	C13—P1—C1	112.27 (19)
H27A—C27—H27B	109.5	C13—P1—C19	109.13 (19)
O2—C27—H27C	109.5	C1—P1—C19	113.18 (19)
H27A—C27—H27C	109.5	C13—P1—C7	111.24 (19)
H27B—C27—H27C	109.5	C1—P1—C7	104.86 (17)
C33—C28—C29	119.1 (4)	C19—P1—C7	105.94 (18)
C33—C28—P2	119.9 (3)	C28—P2—C46	108.42 (18)
C29—C28—P2	120.8 (3)	C28—P2—C34	107.57 (19)
C30—C29—C28	120.3 (4)	C46—P2—C34	108.02 (17)
C30—C29—H29	119.8	C28—P2—C40	114.50 (18)
C28—C29—H29	119.8	C46—P2—C40	111.87 (19)
C31—C30—C29	120.0 (4)	C34—P2—C40	106.16 (18)
C31—C30—H30	120.0	O6—S1—O7	115.7 (3)
C29—C30—H30	120.0	O6—S1—O5	115.9 (3)
C30—C31—C32	120.4 (5)	O7—S1—O5	113.5 (3)
C30—C31—H31	119.8	O6—S1—C55	102.9 (3)
C32—C31—H31	119.8	O7—S1—C55	104.1 (3)
C31—C32—C33	120.7 (5)	O5—S1—C55	102.2 (2)
C31—C32—H32	119.6	O9—S2—O8	114.9 (4)
C33—C32—H32	119.6	O9—S2—O10	112.2 (4)
C28—C33—C32	119.5 (4)	O8—S2—O10	117.8 (4)
C28—C33—H33	120.3	O9—S2—C56	102.3 (3)
C32—C33—H33	120.3	O8—S2—C56	103.0 (3)
C39—C34—C35	119.7 (4)	O10—S2—C56	104.2 (3)
			
C6—C1—C2—C3	−0.4 (6)	C48—C49—C50—C51	−0.4 (7)
P1—C1—C2—C3	−175.8 (3)	C49—C50—C51—O4	179.0 (4)
C1—C2—C3—C4	0.1 (7)	C49—C50—C51—C52	−0.3 (7)
C2—C3—C4—C5	−0.6 (8)	O4—C51—C52—C53	−178.9 (5)
C3—C4—C5—C6	1.4 (7)	C50—C51—C52—C53	0.5 (8)
C4—C5—C6—C1	−1.7 (7)	C51—C52—C53—C48	0.1 (8)
C2—C1—C6—C5	1.2 (6)	C49—C48—C53—C52	−0.7 (7)
P1—C1—C6—C5	176.9 (3)	C47—C48—C53—C52	178.0 (4)
C12—C7—C8—C9	−1.5 (7)	C25—C24—O2—C27	−5.1 (7)
P1—C7—C8—C9	177.4 (3)	C23—C24—O2—C27	176.0 (5)
C7—C8—C9—C10	−0.3 (7)	C50—C51—O4—C54	−0.3 (8)
C8—C9—C10—C11	2.5 (8)	C52—C51—O4—C54	179.0 (5)
C9—C10—C11—C12	−3.0 (9)	C18—C13—P1—C1	−5.4 (4)
C8—C7—C12—C11	1.1 (7)	C14—C13—P1—C1	173.9 (3)
P1—C7—C12—C11	−177.8 (4)	C18—C13—P1—C19	−131.8 (3)
C10—C11—C12—C7	1.2 (9)	C14—C13—P1—C19	47.5 (4)
C18—C13—C14—C15	−0.4 (7)	C18—C13—P1—C7	111.7 (4)
P1—C13—C14—C15	−179.7 (4)	C14—C13—P1—C7	−69.0 (4)
C13—C14—C15—C16	0.2 (8)	C2—C1—P1—C13	−117.5 (3)
C14—C15—C16—C17	−0.8 (9)	C6—C1—P1—C13	66.9 (3)
C15—C16—C17—C18	1.6 (8)	C2—C1—P1—C19	6.6 (4)
C16—C17—C18—C13	−1.8 (7)	C6—C1—P1—C19	−169.0 (3)
C14—C13—C18—C17	1.2 (7)	C2—C1—P1—C7	121.6 (3)
P1—C13—C18—C17	−179.5 (3)	C6—C1—P1—C7	−54.0 (3)
P1—C19—C20—O1	15.7 (5)	C20—C19—P1—C13	55.0 (3)
P1—C19—C20—C21	−164.4 (3)	C20—C19—P1—C1	−70.8 (3)
O1—C20—C21—C26	−10.1 (6)	C20—C19—P1—C7	174.8 (3)
C19—C20—C21—C26	170.1 (4)	C12—C7—P1—C13	3.7 (5)
O1—C20—C21—C22	169.4 (4)	C8—C7—P1—C13	−175.2 (3)
C19—C20—C21—C22	−10.5 (6)	C12—C7—P1—C1	125.3 (4)
C26—C21—C22—C23	0.0 (6)	C8—C7—P1—C1	−53.6 (4)
C20—C21—C22—C23	−179.5 (4)	C12—C7—P1—C19	−114.8 (4)
C21—C22—C23—C24	−0.7 (7)	C8—C7—P1—C19	66.4 (4)
C22—C23—C24—O2	180.0 (4)	C33—C28—P2—C46	160.1 (3)
C22—C23—C24—C25	1.1 (7)	C29—C28—P2—C46	−25.3 (4)
O2—C24—C25—C26	−179.6 (4)	C33—C28—P2—C34	−83.3 (4)
C23—C24—C25—C26	−0.8 (7)	C29—C28—P2—C34	91.3 (4)
C24—C25—C26—C21	0.1 (7)	C33—C28—P2—C40	34.5 (4)
C22—C21—C26—C25	0.3 (6)	C29—C28—P2—C40	−151.0 (3)
C20—C21—C26—C25	179.8 (4)	C47—C46—P2—C28	−53.4 (3)
C33—C28—C29—C30	1.3 (7)	C47—C46—P2—C34	−169.7 (3)
P2—C28—C29—C30	−173.3 (4)	C47—C46—P2—C40	73.8 (3)
C28—C29—C30—C31	−1.5 (8)	C39—C34—P2—C28	160.9 (3)
C29—C30—C31—C32	1.0 (8)	C35—C34—P2—C28	−19.6 (4)
C30—C31—C32—C33	−0.3 (9)	C39—C34—P2—C46	−82.2 (4)
C29—C28—C33—C32	−0.6 (7)	C35—C34—P2—C46	97.3 (4)
P2—C28—C33—C32	174.0 (4)	C39—C34—P2—C40	37.9 (4)
C31—C32—C33—C28	0.2 (8)	C35—C34—P2—C40	−142.6 (3)
C39—C34—C35—C36	−1.2 (7)	C45—C40—P2—C28	114.2 (4)
P2—C34—C35—C36	179.2 (4)	C41—C40—P2—C28	−70.3 (4)
C34—C35—C36—C37	−0.5 (9)	C45—C40—P2—C46	−9.7 (5)
C35—C36—C37—C38	1.6 (10)	C41—C40—P2—C46	165.8 (3)
C36—C37—C38—C39	−0.8 (10)	C45—C40—P2—C34	−127.3 (4)
C35—C34—C39—C38	1.9 (7)	C41—C40—P2—C34	48.2 (4)
P2—C34—C39—C38	−178.5 (4)	F3—C55—S1—O6	55.2 (5)
C37—C38—C39—C34	−0.9 (9)	F2—C55—S1—O6	−65.4 (4)
C45—C40—C41—C42	−1.0 (7)	F1—C55—S1—O6	175.1 (4)
P2—C40—C41—C42	−176.7 (4)	F3—C55—S1—O7	−65.9 (5)
C40—C41—C42—C43	0.9 (8)	F2—C55—S1—O7	173.5 (4)
C41—C42—C43—C44	−0.5 (9)	F1—C55—S1—O7	54.0 (4)
C42—C43—C44—C45	0.2 (9)	F3—C55—S1—O5	175.7 (4)
C41—C40—C45—C44	0.7 (7)	F2—C55—S1—O5	55.1 (4)
P2—C40—C45—C44	176.2 (4)	F1—C55—S1—O5	−64.4 (4)
C43—C44—C45—C40	−0.3 (9)	F5—C56—S2—O9	−53.1 (7)
P2—C46—C47—O3	−19.7 (5)	F4—C56—S2—O9	68.9 (5)
P2—C46—C47—C48	159.4 (3)	F6—C56—S2—O9	−173.7 (5)
O3—C47—C48—C53	6.2 (6)	F5—C56—S2—O8	−172.5 (6)
C46—C47—C48—C53	−172.8 (4)	F4—C56—S2—O8	−50.5 (6)
O3—C47—C48—C49	−175.2 (4)	F6—C56—S2—O8	66.8 (6)
C46—C47—C48—C49	5.8 (6)	F5—C56—S2—O10	64.0 (6)
C53—C48—C49—C50	0.9 (7)	F4—C56—S2—O10	−174.0 (5)
C47—C48—C49—C50	−177.7 (4)	F6—C56—S2—O10	−56.7 (6)

### Hydrogen-bond geometry (Å, °)

**Table:**

*D*—H···*A*	*D*—H	H···*A*	*D*···*A*	*D*—H···*A*
C19—H19A···O5	0.97	2.34	3.207 (6)	149
C19—H19B···O3^i^	0.97	2.54	3.487 (5)	166
C33—H33···O6^ii^	0.93	2.34	3.130 (6)	143
C45—H45···O3	0.93	2.52	3.071 (6)	118
C46—H46A···O1	0.97	2.48	3.417 (5)	162
C46—H46B···O10^iii^	0.97	2.19	3.137 (7)	165
C44—H44···Cg4^iv^	0.93	2.83	3.640 (6)	147

Symmetry codes: (i) *x*−1, *y*, *z*; (ii) −*x*+1, *y*−1/2, −*z*+3/2; (iii) *x*, −*y*+3/2, *z*+1/2; (iv) *x*+1, *y*, *z*.
